# A nationwide cross-sectional survey on hepatitis B and C screening among workers in Japan

**DOI:** 10.1038/s41598-020-68021-2

**Published:** 2020-07-10

**Authors:** Masayuki Tatemichi, Hiroyuki Furuya, Satsue Nagahama, Norihide Takaya, Yukari Shida, Kota Fukai, Satoshi Owada, Hitoshi Endo, Takaaki Kinoue, Masaaki Korenaga

**Affiliations:** 1 Department of Preventive Medicine, School of Medicine, Tokai University, 143 Shimokasuya, Isehara, Kanagawa 259-1193 Japan; 2 All Japan Labor Welfare Foundation, Shinagawa, Tokyo Japan; 3 Medical Corporation Doyukai, Bunkyo, Tokyo Japan; 4 Hepatitis Information Centre, Research Centre for Hepatitis and Immunology, National Centre for Global Health and Medicine, Ichikawa, Chiba Japan

**Keywords:** Gastroenterology, Health care

## Abstract

In Japan, there is no publicly funded screening for hepatitis B virus (HBV) and hepatitis C virus (HCV) infections (using HBs antigen and HCV antibody, respectively) among workers, and workplace health programmes play a crucial role in reducing viral hepatitis-related deaths. The national number of hepatitis screening tests conducted in the workplace is unknown. To provide baseline data for policy formulation, we conducted a nationwide survey to estimate these parameters using data from approximately 10.5 million workers (6.8 million men and 3.8 million women) who underwent mandatory health examinations in their workplaces between April 2016 and March 2017. Among these workers, 494,303 (5.23%, 95% confidence interval [CI] 5.22%–5.24%) and 313, 193 (3.82%, 95% CI 3.81%–3.84%) were screened for HBV and HCV, respectively. Among those who were screened, 0.28% (95% CI 0.27–0.30%) and 0.35% (95% CI 0.33–0.37%) tested positive for HBs antigen and HCV antibody, respectively. According to the age-specific prevalence from the survey an estimated 0.30 and 0.14 million workers in Japan require treatment for HBV and HCV, respectively. To reduce viral hepatitis-related deaths by efficiently identifying workers who need treatment and promoting access to treatment, one-time hepatitis screening of all workers should be considered.

## Introduction

Chronic hepatitis B virus (HBV) and hepatitis C virus (HCV) infection is a major cause of hepatitis-related deaths, such as those due to liver cirrhosis and hepatocellular carcinoma (HCC)^1^. Globally, it is estimated that 257 and 71 million persons were infected by HBV and HCV in 2015, respectively^2^. The control and elimination of these viral infections are the most important public health concerns for the prevention of new HCC cases, particularly in the Western Pacific Region, including Japan^3^.

In Japan, elimination of HBV and HCV infections is regarded as a national priority, and it was estimated that there were approximately 2.1–2.8 million carriers in 2011 based on blood donation and health examination data^4,5^. Further, the number of undiagnosed carriers was estimated to be 0.78 million (HBV: 0.48 million, HCV: 0.30 million), and the number of carriers not in care (i.e. those who had not been diagnosed or who had discontinued medication, despite being carriers of viral hepatitis) was estimated to be 0.50–1.25 million^4,5^. Recently, the treatment for HBV and HCV has dramatically improved through the introduction of nucleoside analogues and direct-acting antiviral agents^6,7^. Thus, to reduce viral hepatitis-related deaths in Japan, it is important to plan policies on how to make the screening for HBV and HCV infection widely accessible to the public and to refer those who screen positive to appropriate medical specialists^8^.

Since 2010, screening of the population for HBV and HCV infections has been promoted by law at a national level in terms of the Basic Act on Hepatitis Measures (part of the Health Promotion Act) and by local government projects (the Countermeasure Projects for Specific Infectious Disease)^9–11^. There are three main types of health insurance in Japan: Union Health Insurance for workers of large companies, Japan Health Insurance Association for workers of small and medium-sized enterprises, and National Health Insurance (covering the general population) for unemployed workers. The government’s policy is to subsidise National Health Insurance to test individuals who have never undergone screening for viral hepatitis at the age of 40 years and every 5 years thereafter and to test those over the age of 40 years who have had liver function abnormalities detected in ‘specific medical check-ups’ annually. HBV and HCV testing is subsidised under the Health Promotion Act, regardless of viral hepatitis testing history^10^. The Countermeasure Projects for Specific Infectious Disease have been implemented in government-designated cities, using health centres and contracted medical institutions to test those of all ages who wish to be tested for hepatitis^11^. As a result, approximately 13 million residents have undergone hepatitis screening^5^.

Although subsidised testing is available at a community level, many workers have undergone mandatory health examinations which do not include screening for viral hepatitis, so they are unwilling to visit a health centre for viral hepatitis screening alone. In addition, there is no national subsidy for workers covered by Union Health Insurance or the Japan Health Insurance Association, and as a result, many workers do not have any healthcare cover for viral hepatitis screening. The government does not subsidise screening of general workers for viral hepatitis, and the coverage of screening is at the discretion of each health insurance union and company. Currently, follow-up examinations and treatment are subsidised, and these subsidies cover 99% of the medical cost of people with viral hepatitis. However, the lack of opportunity for screening for HBV and HCV is a major barrier to accessing treatment because there are no special support measures in workplaces. And then the actual status of screening for HBV and HCV in the workplace is largely unknown.

Several studies have investigated deterrents to HBV and HCV screening among workers. There are several reasons why hepatitis testing has not progressed in Japanese workplaces. Some workers misunderstand that it is possible to infect others via daily social contact, leading to prejudice toward infected workers^12–14^. Moreover, it is possible that employers could refrain from hiring persons with viral hepatitis. The government has now mandated by law that viral hepatitis screening should be carefully performed during mandatory health examinations^15^. Thus, it has resulted in a variety of problems in workplaces that lack medical staff, including how to obtain informed consent for testing, how to manage the test results, determining the person responsible for referring employees found to be positive for viral hepatitis.

In addition to employers, health insurance associations in Japan support screening of workers for HBV and HCV infection, by paying for the tests as a component of their healthcare coverage. However, the decision whether to subsidise these tests is at the discretion of the respective health insurance association.

There is a need to establish a workplace-based viral hepatitis screening programme to assist individuals who are positive for HBV and HCV infection to easily access medical care and follow-up, to provide the full package of counselling and health education, and to promote referral^5^. A pilot study demonstrated that screening for the hepatitis virus among employees encouraged those who screened positive to receive medical care^16^. However, there has never been a nationwide workplace survey of screening practices for viral hepatitis. Thus, this study aimed to estimate the number of workers undergoing screening for HBV and HCV and the prevalence of viral hepatitis among workers at a national level. We also sought to estimate the numbers of HBV and HCV carriers among workers to be used as baseline data for future health policies.

## Subjects and methods

National Federation of Industrial Health Organization (NFIHO) currently contracts with 121 occupational health organisations (or institutions) in 43 out of 47 prefectures in Japan that were eligible for obtaining data on screening and positive rates. NFIHO is the largest group of occupational health organisations that conducts mandatory health examinations, health guidance, and industrial health improvement guidance, based on the Japanese Industrial Health and Safety laws^17^. NFIHO also plays an important role in quality control of medical tests in over 46 million workers among 56 million workers in Japan, including mandatory annual health examinations of 14 million individuals.

We sent a request to all occupational health organisations and institutions affiliated with NFIHO. Data were collected by the institutions during mandatory health examinations, including comprehensive health examinations that were provided in the workplace between April 2016 and March 2017. The institutions provided the following data: number of workers who had undergone the HBV and HCV screening, number of workers who tested positive for HBV and HCV, age (≥ 40 years or not), sex, and type of health insurance.

In this study, on the basis of kinds of data that the institutions recorded, their computer systems and the availability of human resources to tabulate data, eligible institutions were divided according to three criteria: (i) The availability of data on the number of workers screened for HBV and HCV; (ii) The availability of data on the number of workers screened for HBV and HCV according to type of health insurance; and (iii) the number and proportion of tests that were positive. Data were collected on workers of all ages. Data were stratified by age ≥ 40 years or < 40 years in order to compare with data on the general population aged ≥ 40 years available in government reports^18^.

In addition to age, we collected data to stratify the health insurance type (Union Health Insurance for employees working in large companies and Japan Health Insurance Association for employees working in small- or mid-sized companies).

To determine the positive rate of HBV and HCV infection, we collected the positive and negative data of the hepatitis virus tests among workers in all age groups or those aged ≥ 40 years old. Furthermore, to ascertain the detailed age-specific positive rates, we conducted the survey with the cooperation of two large institutions affiliated with NFIHO (All Japan Labor Welfare Foundation and Medical Corporation Doyukai). These occupational health organisations are located around the Kanto area of Japan.

This project was approved by the Tokai University clinical research review board (16R-076). This study involved a retrospective analysis of secondary data and did not collect personally identifiable information. As we did not collect any personally identifiable information, on the basis of existing regulations in Japan, personal informed consent was not required.

### Analysis methods

The data provided by each occupational health institute were collected at once. The screening rate and positive rate were calculated using the number of examinees and positive results, respectively, as the numerator and the health examinations as the denominator. The 95% confidence intervals (CIs) were calculated using the Agresti-Coull method.

In order to compare the data among testing in the general population at a local government level and workplace-based testing, the results of the viral hepatitis screening tests performed as per the Health Promotion Act in 2016^18^ were used as a reference (Supplement 2).

To estimate the number of workers who would require treatment for viral hepatitis, the data on the number of workers released by the Labour Force Survey of Japan in 2016^19^ were used as a reference. For HCV, given that the screening for HCV is an antibody-based test, the positive results also include cases that were infected and cured in the past. According to previous reports^20–22^, approximately 50% of individuals who test positive for HCV antibody have past infection, not chronic infection; therefore, the number of workers aged under 65 years who are positive for hepatitis B surface (HBs) antigen or the number of workers aged under 65 years who require treatment for HCV infection was estimated as follows:

the estimated number of workers who are positive for HBs antigen.

$$= {\sum }_{i=2}^{6}(Ni\times Pi)$$

the estimated number of workers who require treatment for HCV infection.

$$= \frac{1}{2}{\sum }_{i=2}^{6}(Ni\times Pi)$$

 

*Ni* = number of workers in the age group *i*; *Pi* = proportion of workers who are positive in the age group *i.*

*i* = 2 (20–29 years), *i* = 3 (30–39 years), *i* = 4 (40–49 years),*i* = 5 (50–59 years), *i* = 6 (60–69 years).

(*N*_6 =_ number of workers in the 60–64-year age group).

Statistical significance was determined using χ^2^-test (alpha error was set at p < 0.05). We also determined the results as significant when the 95% CIs did not overlap.

## Results

### Participating institutions and data

For the survey of all examinees, 84 out of the 121 NFIHO-associated occupational health organisations or institutions positively responded to our request and participated in the study (Supplement Table S1).

The number of collaborating institutions for each sub-analysis was different because each sub-analysis required detailed tabulation such as positive numbers for HBs antigen and/or HCV antibody. The reasons for non-participation were mainly lack of time, cost of modifying the system to collect data, and that person-hours could not be allocated to aggregate data to verify their correctness.

Finally, data were obtained for 10,541,326 workers, of whom 6,880,992 and 3,880,334 were men and women, respectively. The data obtained were collected from 79 institutions (5 institutions did not provide data according to age) with a total number of 6,010,147 workers (3,771,959 men; 2,238,188 women). Further, 62 institutions provided data that could be tabulated for the calculation of the positive rates.

### Screening rates among the workers

Table 1 shows the number and percentage of workers who were screened for viral hepatitis. Significantly more workers were tested for HBV infection than for HCV infection (p < 0.001 by χ^2^ test) (5.23%, 95% CI 5.22–5.24% vs. 3.83%, 95% CI 3.81–3.84% in all aged workers, 6.20%, 95% CI 6.18–6.22% vs. 4.78%, 95% CI 4.76–4.80% in workers aged ≥ 40 years). Moreover, female workers had significantly higher odds (p < 0.001) to undergo screening tests than male workers (data not shown). The screening rates for HBs antigen among male and female workers of all ages were 4.74%, 95% CI 4.73–4.76% and 6.08%, 95% CI 6.06–6.11%, respectively. The screening rates for HCV antibody among male and female workers were also significantly (p < 0.001) different (3.4%, 95% CI 3.37–3.38% and 4.60%, 95% CI 4.58–4.62%, respectively).

**Table 1:** Proportion of workers undergoing mandatory workplace examination screened for hepatitis B surface (HBs) antigen and hepatitis C virus (HCV) antibody by sex and age (all ages and 40 years or older).

	All ages			Age ≥ 40 years		
	Men	Women	All	Men	Women	All
No. of workers who had mandatory health examinations	6,680,992	3,860,334	10,541,326	3,771,956	2,238,188	6,010,144
No. tested for HBs antigen	316,907	234,806	551,713	222,511	150,270	372,781
Proportion tested for HBs antigen (%)	4.74	6.08	5.23	5.90	6.71	6.20
95% CI (%)	4.73–4.76	6.06–6.11	5.22–5.24	5.87–5.92	6.68–6.75	6.18–6.22
No. tested for HCV antibody	225,776	177,571	403,347	157,848	129,464	287,312
Proportion tested for HCV (%)	3.38	4.60	3.83	4.18	5.78	4.78
95% CI (%)	3.37–3.39	4.58–4.62	3.81–3.84	4.16–4.20	5.75–5.81	4.76–4.80

The results according to the type of health insurance are shown in Table 2. A total of 1,770,235 workers from 42 institutions with Union Health Insurance, and 915,879 workers from 40 institutions with Japan Health Insurance Association insurance were included in the analysis. The HBV and HCV screening rates were significantly (p < 0.001) higher (approximately double) in workers with Union Health Insurance than in those with Japan Health Insurance Association insurance (9.78% vs. 3.97% for HBV, 5.92% vs. 3.10% for HCV among workers aged ≥ 40 years).

**Table 2:** Proportion of workers screened for hepatitis B surface (HBs) antigen and hepatitis C virus (HCV) antibody by type of health insurance, sex, and age (all ages and 40 years or older).

	All ages			Age ≥ 40 years		
	Men	Women	All	Men	Women	All
**Union Health Insurance**						
No. who had mandatory health examinations	1,129,553	640,682	1,770,235	661,094	372,664	1,033,758
No. tested for HBs antigen	86,291	51,936	138,227	65,277	35,777	101,054
Proportion tested for HBs antigen (%)	7.64	8.11	7.81	9.87	9.60	9.78
95% CI (%)	7.59–7.69	8.04–8.17	7.77–7.85	9.80–9.94	9.51–9.69	9.72–9.83
No. tested for HCV antibody	52,729	34,561	87,290	38,716	22,487	61,203
Proportion tested for (%)	4.67	5.39	4.93	5.86	6.03	5.92
95% CI (%)	4.63–4.71	5.34–5.45	4.90–4.96	5.80–5.91	5.96–6.11	5.87–5.66
**Japan Health Insurance Association**						
No. who had mandatory health examinations	607,552	308,327	915,879	450,293	225,093	675,386
No. tested for HBs antigen	18,230	15,393	33,623	14,928	11,866	26,794
Proportion tested for HBs antigen (%)	3.00	4.99	3.67	3.32	5.27	3.97
95% CI (%)	2.96–3.04	4.91–5.07	3.63–3.71	3.26–3.36	5.18–5.36	3.92–4.01
No. positive for HCV antibody	14,052	12,916	26,968	11,255	9,856	21,111
Proportion tested for HCV antibody (%)	2.3	4.2	2.9	2.5	4.4	3.1
95% CI (%)	2.28–2.35	4.12–4.26	2.91–2.98	2.45–2.55	4.29–4.46	3.08–3.17

### Positive rates among the workers/residents

Among the participating institutions, test results of 62 organisations were available for tabulation and calculation of the positive rates (Table 3). Among the 494,303 workers who underwent HBs antigen testing (284,034 men and 210,269 women) and 315,193 workers who underwent HCV antibody testing (172,037 men and 143,156 women), the rates of workers aged ≥ 40 years who tested positive for HBs and HCV antibody were 0.35% (95% CI 0.33–0.37%) and 0.44% (95 CI 0.41–0.46%), respectively. Regarding the HBs antigen, the positive rate was not significantly different between men and women (0.29%, 95% CI 0.27–0.31 vs. 0.27%, 95% CI 0.25–0.29). Regarding HCV antibody, the positive rate among workers aged ≥ 40 years was significantly (p < 0.001) higher in men than in women (0.51%, 95% CI 0.47–0.55% vs. 0.35%, 95% CI 0.32–0.39%).

**Table 3:** Proportion of workers positive for hepatitis B surface (HBs) antigen and hepatitis C virus (HCV) antibody by sex and age (all ages and 40 years or older).

	All ages			Age ≥ 40 years		
	Men	Women	All	Men	Women	All
**HBs antigen**						
No. tested	284,034	210,269	494,303	202,351	136,030	338,381
No. positive	835	562	1,397	714	461	1,175
Positive rate (%)	0.29	0.27	0.28	0.35	0.34	0.35
95% CI (%)	0.27–0.31	0.25–0.29	0.27–0.30	0.33–0.38	0.31–0.37	0.33–0.37
**HCV antibody**						
No. tested	172,037	143,156	315,193	122,203	108,550	230,753
No. positive	672	419	1,091	622	382	1,004
Positive rate (%)	0.39	0.29	0.35	0.51	0.35	0.44
95% CI (%)	0.36–0.42	0.26–0.32	0.33–0.37	0.47–0.55	0.32–0.39	0.41–0.46

According to the government reports for the Health Promotion Act in 2016^17^, the number of residents aged ≥ 40 years undergoing viral hepatitis screening was 728,131 and 728,684 for HBV and HCV; 0.65% and 0.32%, respectively. Thus, the positive rate for HBV was significantly (as per confidence intervals) lower in workers than in the resident individuals. On the other hand, the positive rate for HCV was significantly higher in workers than in residents.

### Age-specific positive rates of viral hepatitis among the workers and the general population

Tables 4 and 5 show the HBV and HCV screening results according to the age and sex. The prevalence of chronic HBV and HCV seropositivity was very low among workers aged < 30 years, and the prevalence increased with advancing age. Among male workers aged > 60 years, the positive rates for both HBV and HCV infections were approximately 1%. However, according to the government reports for the Health Promotion Act in 2016^18^, the prevalence of hepatitis B by age was 0.4–0.5% in residents aged 40–49 years, 0.6–0.7% in those aged 50–59 years, and 0.8% in those aged 60–69 years (Table 4)^18^. Similarly, in the case of HCV, the positive rate among the residents was 0.1–0.2%, 0.3%, and 0.3%, respectively (Table 5)^18^. The prevalence of HBV and HCV according to government reports is also shown in the tables.

**Table 4:** Proportion of workers screened for and infected with hepatitis B virus by sex and age group.

	Age (years)				
	20–29	30–39	40–49	50–59	60–69
**Men**					
No. tested for HBs antigen	1,207	5,959	13,680	11,937	7,790
No. positive for HBs antigen	0	17	82	79	88
Positive rate for HBs antigen (%)	0.00	0.29	0.60	0.66	1.13
95% CI (%)	0–0.22	0.14–0.43	0.47–0.73	0.51–0.81	0.89–1.37
**Women**					
No. tested for HBs antigen	1,058	4,118	8,412	6,395	3,379
No. positive for HBs antigen	1	13	56	31	23
Positive rate for HBs antigen (%)	0.09	0.32	0.67	0.48	0.68
95% CI (%)	0–0.41	-0.07–0.41	0.49–0.84	0.31–0.66	0.39–0.97
**All**					
No. tested for HBs antigen	2,265	10,077	22,092	18,332	11,169
No. positive for HBs antigen	1	30	138	110	111
Positive rate for HBs antigen (%)	0.04	0.30	0.62	0.60	0.99
95% CI (%)	0–0.19	0.19–0.41	0.52–0.73	0.49–0.71	0.81–1.12
Government report in 2016 (Ref.^18^) N = 728,131	–	–	0.4–0.6%	0.6–0.7%	0.8%

**Table 5:** Proportion of workers screened for and seropositive for hepatitis C virus (HCV) by sex and age group.

	Age (years)				
	20–29	30–39	40–49	50–59	60–69
**Men**					
Number tested for HCV	1,132	5,428	12,470	10,882	7,220
Number positive for HCV	1	10	51	97	71
Positive rate for HCV (%)	0.09	0.18	0.41	0.89	0.98
95% CI (%)	0–0.38	0.06–0.31	0.29–0.52	0.71–1.07	0.75–1.21
**Women**					
Number tested for HCV	883	3,761	7,583	5,758	3,110
Number positive for HCV	3	11	17	40	25
Positive rate for HCV (%)	0.34	0.29	0.22	0.69	0.80
95% CI (%)	0–082	0.11–0.48	0.11–0.34	0.48–0.91	0.48–1.13
**All**					
Number tested for HCV	2,015	9,189	20,053	16,640	10,330
Number positive for HCV	4	21	68	137	96
Positive rate for HCV (%)	0.20	0.23	0.34	0.82	0.93
95% CI (%)	0–0.43	0.13–0.33	0.24–0.40	0.69–0.96	0.74–1.12
Government report in 2016 (Ref.^18^) (N = 728,684)	–	–	0.1–0.2%	0.3%	0.3%

### The estimation of number of workers requiring treatment

According to the Labour Force Survey of Japan^19^, the numbers of workers in 2016 were as follows: 10.10 million aged 20–29 years, 12.90 million aged 30–39 years, 16.27 million aged 40–49 years, 12.97 million aged 50–59 years, and 8.16 million aged 60–64 years. From our results, the positive rates of HBV and HCV were estimated to be 0.04% and 0.20% among those aged 20–29 years, 0.30% and 0.23% among those aged 30–39 years, 0.62% and 0.34% among those aged 40–49 years, 0.60% and 0.82% among those aged 50–59 years, and 0.99% and 0.93% among those aged 60 years and over, respectively (Tables 4 and 5). Thus, an estimated 0.30 million workers have chronic HBV infection, and 0.14 million workers require treatment for HCV infection.

## Discussion

The results of this (first) nationwide survey in Japanese workplaces revealed that only 5.23% and 3.83% of workers underwent screening for HBV and HCV infection, respectively, through the social system, despite Japanese workers being required by law to undergo blood examinations at 35 years of age and then after 40 years of age^17^. Further, the government in its Basic Act on Hepatitis Measures^9^ recommends the additional viral hepatitis screening.

Considering a 5% annual screening rate, if different workers undergo these tests every year, then, in the following 10 years, about half of the workers would have undergone viral hepatitis screening, particularly because viral hepatitis screening needs to be performed once in a lifetime. However, in practice, the same items of the annual health check-up may be repeated, and there is high (75%) likelihood that the same worker would undergo a repeat examination (unpublished data); hence, the overall number of workers undergoing hepatitis testing may be limited in practice.

The screening rate was higher for HBV than for HCV among the workers including those older than 40 years. HBV infection is recognised as an occupational exposure risk for health care workers and laboratory technicians^23,24^. Thus, we believe that a high proportion of workers in these occupations received the screening test for HBV in the mandatory health check-up. In addition, workers from large companies that are beneficiary of Union Health Insurance undergo comprehensive health examinations (Ningen-dock) as the mandatory health check-up, primarily including the hepatitis screening test (HBs antigen), but not the HCV, which is optional^25^.

In the analysis of the screening rates for workers stratified by their health insurance type, the screening rate for workers was approximately two times higher in those with Union Health Insurance than in those with Japan Health Insurance Association. This might be due to the difference in the authorisation from health insurance for viral hepatitis screening tests. In particular, as mentioned above, the Union Health Insurance authorises Ningen-dock, primarily including the HBs antigen test^25^. On the other hand, health check-up covered by the Japan Health Insurance Association primarily does not include any mandatory screening tests for hepatitis viruses, but instead they are only optional^26^.

Among workers of all ages and workers aged ≥ 40 years, the screening rate for both HBV and HCV was higher in women than in men. There may be a greater concern about acquiring viral hepatitis among women because they are frequently in contact with blood during menstruation, pregnancy, and childbirth. In the workplace, hepatitis screening is optional, not mandatory, thus the probability of testing is likely to depend in part on the worker’s knowledge and awareness the disease. Therefore, it is considered that information and education on viral hepatitis for workers may be critically important in improving screening rates.

In this study, the prevalence of HBV was negligible among those aged < 30 years, and among those over the age of 30 years, the prevalence increased with age. The prevalence of HBV among those tested in the community^18^ was higher than that of those tested in the workplace. This may be due to the difference in age distribution among the workers and those tested in the community: those tested in the community are likely to have been older than the workers.

In contrast, the HCV antibody prevalence was higher in the workplace than that in the government report (0.44% in workplace vs. 0.32% in residents). Further, the age-specific positive rate was higher in workers than that for residents. This difference could be explained by the subjectivity in test selection for the workers (workers with known hepatitis C status intentionally selecting the test, resulting in higher positive rate), which is not the case with government reports because government reports did not include subjects who had previously undergone the screening tests.

In Japan, the mandatory health examinations are required by the Industrial Safety and Health Act, and the examinations include mainly BMI, blood pressure, liver function, lipid examinations, blood glucose, chest X-ray, urine protein and electrocardiogram, targeting to prevent cerebral-cardio vascular events. Then the blood examinations are required at the mandatory health examinations when the applicants are hired in a company and at the age of 35 years and ≥ 40 years^17^; thus, it would be desirable to conduct a viral hepatitis screening test together with the mandatory health examinations. On the basis of our results, approximately 100,000–200,000 workers require treatment for HBV and HCV combined. To reduce viral hepatitis-related deaths, the Japanese government should consider subsidising hepatitis screening for employees, ensure proper conduct of screening tests, and encourage consultation of positive cases with an appropriate medical specialist.

The strength of this study is that the actual number of hepatitis screening tests conducted at the national level including 10 million workers was analysed because the primary purpose of this study was to clarify the actual screening rate of viral hepatitis among workers. Thus, this study surveyed the actual number of hepatitis tests conducted from health check-up organisations affiliated with the NFIHO, which are associations with a substantial population of workers. In this study, therefore, the selection bias is considered to be small because approximately 75% of the 15 million individuals who had undergone mandatory health examinations by the NFIHO Japan were included. The reason why some institutes did not participate in this survey seems to be unrelated to hepatitis screening tests.

The limitations of this study are that it is a cross-sectional survey, and there is no precise information on the duplication of testing for the same worker. It could be possible that the number of examinees includes repeated measurements for a worker. In Japan, there is currently no unique ID continued over a lifetime for medical uses, although the government is considering creating one. The insurance number can be used as a unique ID, but if a worker leaves the company and goes to work for another company, the ID changes, so it is not reliable as an identifier. Although, the number of people who have taken the test multiple times during the two-year study period is likely to have been limited, we will plan to investigate repeat testing in a future study. Secondly the data on positive rates could have some bias. In particular, interpretation of the HCV data may be problematic because HCV-positive workers could intentionally seek HCV antibody testing. Therefore, our figures of HCV prevalence among the workers might be overestimated. Despite these limitations, we believe that our results particularly contribute to providing baseline data for future measurements in Japan.

Given that the prevalence was < 0.5% for workers younger than 50 years, the costs involved in screening such workers with low prevalence may be seen as a barrier. Thus, it seems that the theoretical strategies should focus on high-risk groups, including birth cohort, drug users, or residence area. However, some reports showed that universal screening is effective and the most cost-effective strategy if followed with a rapid initiation of treatment after the diagnosis^27,28^. In Japan, approximately 15% of liver function abnormalities including alanine aminotransferase, aspartate aminotransferase, and gamma-glutamyl transferase are found in blood tests through mandatory health examinations among workers aged ≥ 40 years^29^. Given that, viral hepatitis screening is indispensable for all of these to make a diagnosis against liver dysfunction; we believe that a one-time universal hepatitis screening test is necessary in the workplace.

We believe that viral hepatitis-related deaths could be efficiently reduced by conducting universal hepatitis screening in the workplace, along with health education regarding hepatitis to eliminate misconceptions and discrimination associated with hepatitis infection.

At present, most cases of HCV can be completely cured with oral drugs, and vertical transmission of HBV has been almost completely prevented by vaccination. If those who become infected with HBV and HCV are screened and treated promptly, these infections do not have adverse long-term health effects. The government should set a goal of screening all workers for viral hepatitis at a time to terminate hepatitis-related deaths.

## Supplementary information

Supplementary information.

## Data Availability

Permission was obtained to use the data in this study by National Federation of Industrial Health Organization, All Japan Labor Welfare Foundation and Medical Corporation Doyukai. The data that support the findings of this study are available on request from the corresponding author [M.T.]. The data are not publicly available due to no approval from the ethics review board.
