# Supplementary material for: A nationwide cross-sectional survey on hepatitis B and C screening among workers in Japan
Source: Sci Rep. 2020 Jul 10;10:11435. doi: 10.1038/s41598-020-68021-2 (PMC7351736; doi:10.1038/s41598-020-68021-2)
Supplement: Supplementary file 1 — Supplementary information. [file 41598_2020_68021_MOESM1_ESM.docx]

**A nationwide cross-sectional survey on hepatitis B and C screening among workers in Japan**

**Masayuki Tatemichi^1)*^, Hiroyuki Furuya^1)^, Satsue Nagahama^2)^, Norihide Takaya^3)^, Yukari Shida^1)^, Kota Fukai^1)^, Satoshi Owada^1)^, Hitoshi Endo^1)^, Takaaki Kinoue^1)^, Masaaki Korenaga^4)^**

1. Department of Preventive Medicine, Tokai University, School of Medicine, Kanagawa, Japan
2. All Japan Labor Welfare Foundation
3. Medical Corporation Doyukai
4. Hepatitis Information Centre, Research Centre for Hepatitis and Immunology　National Centre for Global Health and Medicine

| Supplement Table 1. Participant institutions in this study by 47 prefectures among Japan | | | | |
| --- | --- | --- | --- | --- |
| No | Prefecture | Number of workers (in thousands)^1)^ | Institutions associated with NFHIO^2)^ | Participant institutions in this study |
| 1 | Hokkaido | 2662 | 1 | 1 |
| 2 | Aomori | 673 | 2 | 1 |
| 3 | Iwate | 670 | 1 | 1 |
| 4 | Miyagi | 1198 | 2 | 2 |
| 5 | Akita | 502 | 0 | 0 |
| 6 | Yamagata | 588 | 0 | 0 |
| 7 | Fukushima | 999 | 1 | 1 |
| 8 | Ibaraki | 1518 | 0 | 0 |
| 9 | Tochigi | 1047 | 0 | 0 |
| 10 | Gunma | 1024 | 0 | 0 |
| 11 | Saitama | 3878 | 1 | 1 |
| 12 | Chiba | 3336 | 4 | 2 |
| 13 | Tokyo | 7768 | 23 | 12 |
| 14 | Kanagawa | 4931 | 5 | 5 |
| 15 | Niigata | 1204 | 3 | 2 |
| 16 | Toyama | 571 | 1 | 1 |
| 17 | Ishikawa | 615 | 2 | 1 |
| 18 | Fukui | 432 | 2 | 2 |
| 19 | Yamanashi | 426 | 1 | 0 |
| 20 | Nagano | 1137 | 2 | 1 |
| 21 | Gifu | 1123 | 3 | 2 |
| 22 | Shizuoka | 1992 | 7 | 5 |
| 23 | Aichi | 4015 | 11 | 9 |
| 24 | Mie | 970 | 1 | 1 |
| 25 | Shiga | 716 | 1 | 0 |
| 26 | Kyoto | 1373 | 3 | 2 |
| 27 | Osaka | 4473 | 5 | 1 |
| 28 | Hyogo | 2779 | 6 | 4 |
| 29 | Nara | 657 | 0 | 0 |
| 30 | Wakayama | 493 | 2 | 2 |
| 31 | Tottori | 298 | 1 | 1 |
| 32 | Shimane | 349 | 1 | 1 |
| 33 | Okayama | 963 | 3 | 2 |
| 34 | Hiroshima | 1453 | 4 | 4 |
| 35 | Yamaguchi | 703 | 1 | 0 |
| 36 | Tokushima | 367 | 1 | 0 |
| 37 | Kagawa | 492 | 1 | 1 |
| 38 | Ehime | 688 | 1 | 1 |
| 39 | Kochi | 367 | 0 | 0 |
| 40 | Fukuoka | 2574 | 9 | 7 |
| 41 | Saga | 430 | 1 | 1 |
| 42 | Nagasaki | 686 | 1 | 1 |
| 43 | Kumamoto | 915 | 2 | 1 |
| 44 | Oita | 594 | 1 | 1 |
| 45 | Miyazaki | 558 | 1 | 1 |
| 46 | Kagoshima | 818 | 2 | 2 |
| 47 | Okinawa | 712 | 1 | 1 |
|  | Total | 66737 | 121 | 84 |
| 1) Labour Force Survey (https://www.stat.go.jp/data/roudou/pref/zuhyou/lt02y.xls) (2016) | | | | |
| 2) National Federation of Industrial Health Organization | | |  |  |

Supplement 2 (in English of ref 17)
